# Breaking barriers: Respiratory viral strategies targeting the host's nuclear pore complex and nuclear transport pathways

**DOI:** 10.1091/mbc.E25-07-0322

**Published:** 2025-11-12

**Authors:** Yael Udi, John D. Aitchison, Michael P. Rout, Samson Obado

**Affiliations:** ^a^Laboratory of Cellular and Structural Biology, The Rockefeller University, New York, NY 10065; ^b^Center for Global Infectious Disease Research, Seattle Children's Research Institute, Seattle, WA 98101; ^c^Department of Pediatrics, University of Washington, Seattle, WA 98195; ^d^Department of Biochemistry, University of Washington, Seattle, WA 98105; ETH Zurich

## Abstract

From stealthy infiltrators to blunt-force saboteurs, many human viruses––perhaps all—disrupt nuclear transport to control host gene expression, suppress immune responses, and redirect cellular resources toward their own replication. Among them, respiratory viruses stand out for their global impact and relentless evolution, from seasonal scourges to pandemic threats. Focusing on adenoviruses, influenza, rhinoviruses, RSV, and SARS-CoV-2, we explore a series of molecular case studies that reveal both shared strategies and the diverse molecular innovations these respiratory pathogens use to subvert the nuclear transport machinery. We organize these tactics into six recurring strategies: NPC docking and nuclear entry, inhibition of immune-factor import, hijacking nuclear protein transport and karyopherins, sabotage of host mRNA export, degradation of FG-Nups, and exploitation of mitotic nuclear envelope breakdown. These insights not only illuminate fundamental virus–host conflicts but may also point the way toward new therapeutic vulnerabilities in the viruses’ attack strategies.

## Abstract (executive summary)

- This review highlights a critical aspect of the interface between viruses and host cells, representing a fundamental aspect of molecular cell biology.
- We focus on common respiratory viruses, which have been recognized as being a significant threat to public health in the last 5 years.
- We describe the critical role the nucleus has in viral replication and highlight some representative mechanisms viruses use to access the nucleus and control nuclear transport, thereby subverting the host's immune response and enabling successful viral replication.

## INTRODUCTION

Viruses do not just infect cells; they seize control, bending cellular machinery to serve their own purposes. In doing so, they act as nature's cell biologists—probing, rewiring, and exposing critical cellular processes. Studying these viral strategies not only reveals fundamental insights into cell biology but also exposes vulnerabilities that can be targeted for therapeutic intervention. Among human pathogens, respiratory viruses stand out for both their global impact and the precision with which they manipulate host pathways. Their high transmissibility and evolutionary adaptability have made them perennial public-health threats, from endemic viruses such as rhinoviruses and RSV to pandemic threats like influenza and SARS-CoV-2 (Box 1).

**BOX 1: An introduction to five key respiratory viruses**

**FIGURE 1:**
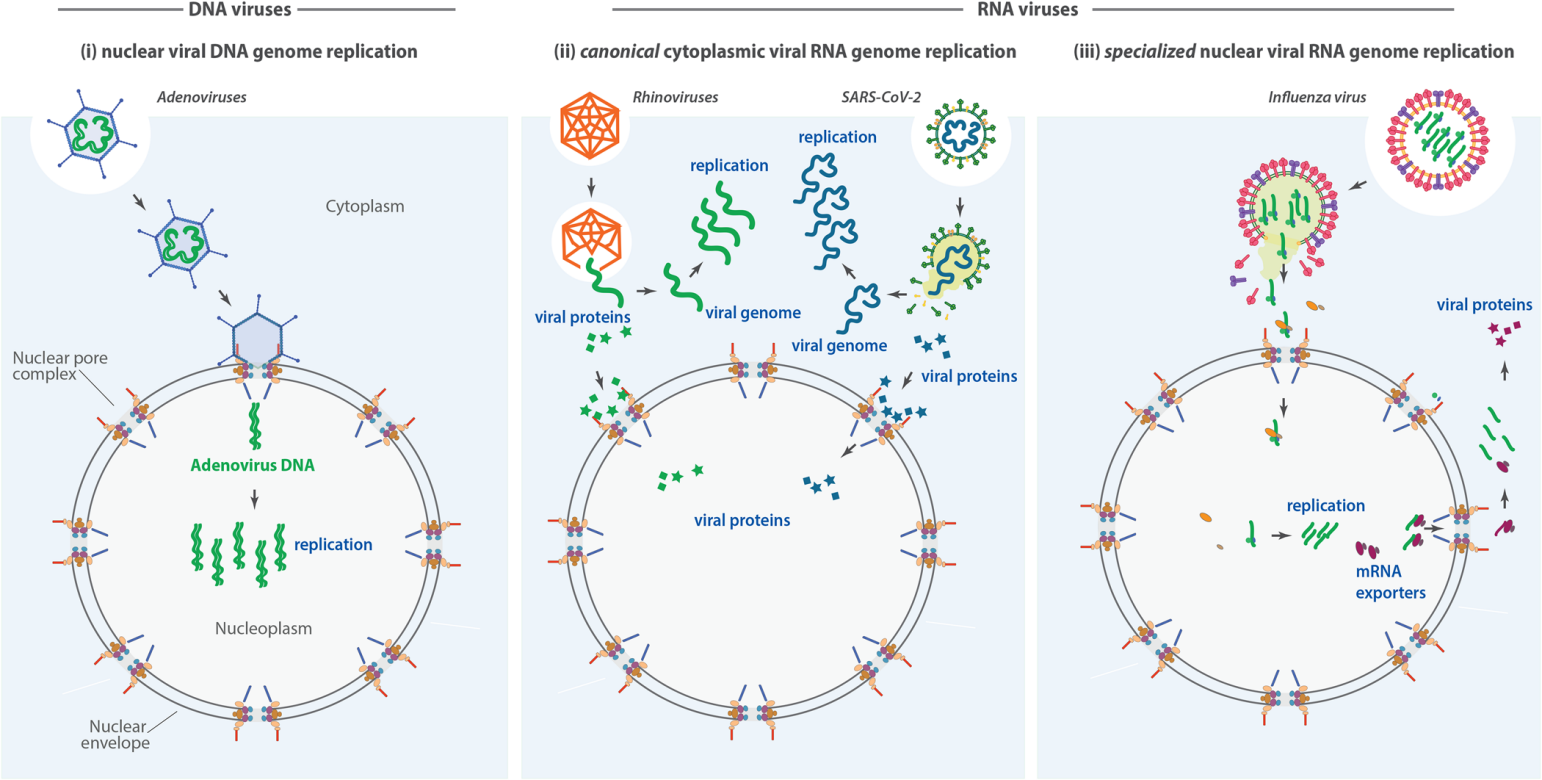
Strategies of viral genome replication. Representative respiratory viruses use the nucleus in different ways to promote their replication. (i) DNA viruses such as adenoviruses deliver their genomes directly into the nucleus, where replication and transcription occur using host or viral machinery. Viral mRNAs are exported through the NPC for translation in the cytoplasm. (ii) Canonical RNA viruses, such as rhinoviruses SARS-CoV-2, replicate entirely in the cytoplasm, bypassing the nucleus. (iii) Distinctive nuclear-replicating RNA viruses, such as the influenza virus, import their RNA genomes into the nucleus to access host replication and transcription machinery. Viral mRNAs produced in the nucleus are exported via NPCs for cytoplasmic translation. The figure highlights key cellular compartments and pathways used by viruses, including NPCs, the nuclear envelope, and viral protein localization.

This review focuses on adenoviruses, rhinoviruses, influenza virus, respiratory syncytial virus (RSV), and severe acute respiratory syndrome coronavirus 2 (SARS-CoV-2) (Figure 1), causative agents of a wide range of respiratory diseases that are among the most common and concerning respiratory viruses in circulation today. Nevertheless, they also serve as exemplars of the general strategies viruses use to usurp nucleocytoplasmic transport.

**Human adenoviruses** (HAdV) cause a wide range of illnesses in humans (MacNeil *et al*., 2023). HAdVs are large nonenveloped double-stranded DNA viruses, whose ∼35kb genome encoding ∼40 different viral proteins are contained within a ∼150 MDa icosahedral capsid that is ∼90 nm in size (Nemerow *et al*., 2012). The icosahedral capsid shell is composed of three major capsid proteins (hexon, penton, and fiber), and minor cement proteins (IIIa, VI, VIII, and IX) connecting the major structural units with each other and the viral core, the latter containing the viral genome and four core proteins (V, VII, µ, and terminal protein) (Ahi and Mittal, 2016). As a DNA virus, the hAdV genome needs to overcome the permeability barrier of the NPC to enter the nucleus in order to replicate and propagate (Figures 1 and 3).

**Rhinoviruses** are the prevalent cause of the common cold. Rhinoviruses are members of the Picornaviridae, small, nonenveloped RNA viruses with a ∼8 kb–positive sense RNA genome that is encased in an icosahedral capsid (Rossmann *et al*., 1985). As is typical of most RNA viruses, Picornaviruses do not require entry into the nucleus for replication. The picornaviral genome that has a 5′-UTR and a 3′ poly A tail is translated in the cytoplasm by host ribosomes into a polyprotein. The 5′end of the genome encodes structural capsid proteins, while the 3′end encodes nonstructural proteins involved in viral replication (Zell, 2018). The polyprotein is cotranslationally and post-translationally processed into individual components by two proteases, 2A (2Apro) and 3C (3Cpro). 2Apro makes the initial cleavage during translation to separate all the viral coat proteins (VP1-4) (Zell, 2018), while 3Cpro makes all subsequent polyprotein cleavages (Figure 1). These two proteases have been implicated in the degradation of NPC components and inhibition of nucleocytoplasmic transport. Thus, despite not requiring direct access to the nucleus to propagate, shutting down host NPC components is critical to a successful viral infection (Figure 3).

**Influenza viruses** are RNA viruses that belong to the Orthomyxoviridae family (Paules and Subbarao, 2017). The influenza viral genome consists of eight segmented negative-sense single-stranded RNAs encoding 10 core polyproteins: the RNA-dependent RNA polymerase subunits polymerase basic 1 (PB1), polymerase basic 2 (PB2), and polymerase acidic (PA), the glycoproteins HA and NA, NP, the matrix protein M1, the M2 ion channel, the nonstructural protein NS1, and, the nuclear export protein (NEP) (previously termed NS2) (Staller and Barclay, 2021). Each RNA segment is packaged into a viral ribonucleoprotein (vRNP) complex together with a heterotrimeric viral RNA-dependent RNA polymerase. The RNA polymerase is a heterotrimer formed by three viral RNA polymerase subunits, PB1, PB2 PA, which associate with multiple copies of NPs (Jakubiec *et al*., 2006; Te Velthuis *et al*., 2021) (Figure 1). NP, PB1, PB2, and PA all contain NLSs and exploit the importin α/β pathways for nuclear import (Ozawa *et al*., 2007; Nguyen *et al*., 2023) (Figure 3).

**RSV** is a major cause of lower tract respiratory disease, with infants, the elderly, and immunocompromised individuals being particularly susceptible. RSV is a negative-sense, single-stranded RNA virus whose genome encodes 11 proteins: nine structural and two nonstructural. The structural proteins include the envelope glycoproteins F, G, and SH; the nucleocapsid proteins N, P, and L; the nucleocapsid-associated proteins M2-1 and M2-2; and the matrix protein M. The two nonstructural proteins, NS1 and NS2, play key roles in antagonizing host immune responses (Borchers *et al.*, 2013). M protein is a major structural protein within the virion and has a key role in RSV assembly (Figure 1). During RSV infection, the viral nonstructural proteins NS1 and NS2 play key roles in manipulating the host immune response (Spann *et al.*, 2004) (Figure 3).

**SARS-CoV-2** is a single-stranded, positive-sense RNA virus that replicates primarily in the epithelial cells of the respiratory tract (V'Kovski *et al*., 2021; Jackson *et al*., 2022). The SARS-CoV-2 genome is ∼30 kb in size, representing the largest known single stranded viral RNA genome, and encodes four structural proteins (spike, membrane, envelope, and NP) as well as 16 nonstructural proteins (Nsps) and an additional nine predicted accessory proteins (Orfs 3a, 3d, 6, 7a, 7b, 8, 9b, 9c, and 10) (Astuti and Ysrafil, 2020; Naqvi *et al*., 2020) (Figure 1). Orf6 has been shown to subvert NPC function through interactions with several Nups and transport factors (Figure 3).

**FIGURE 2:**
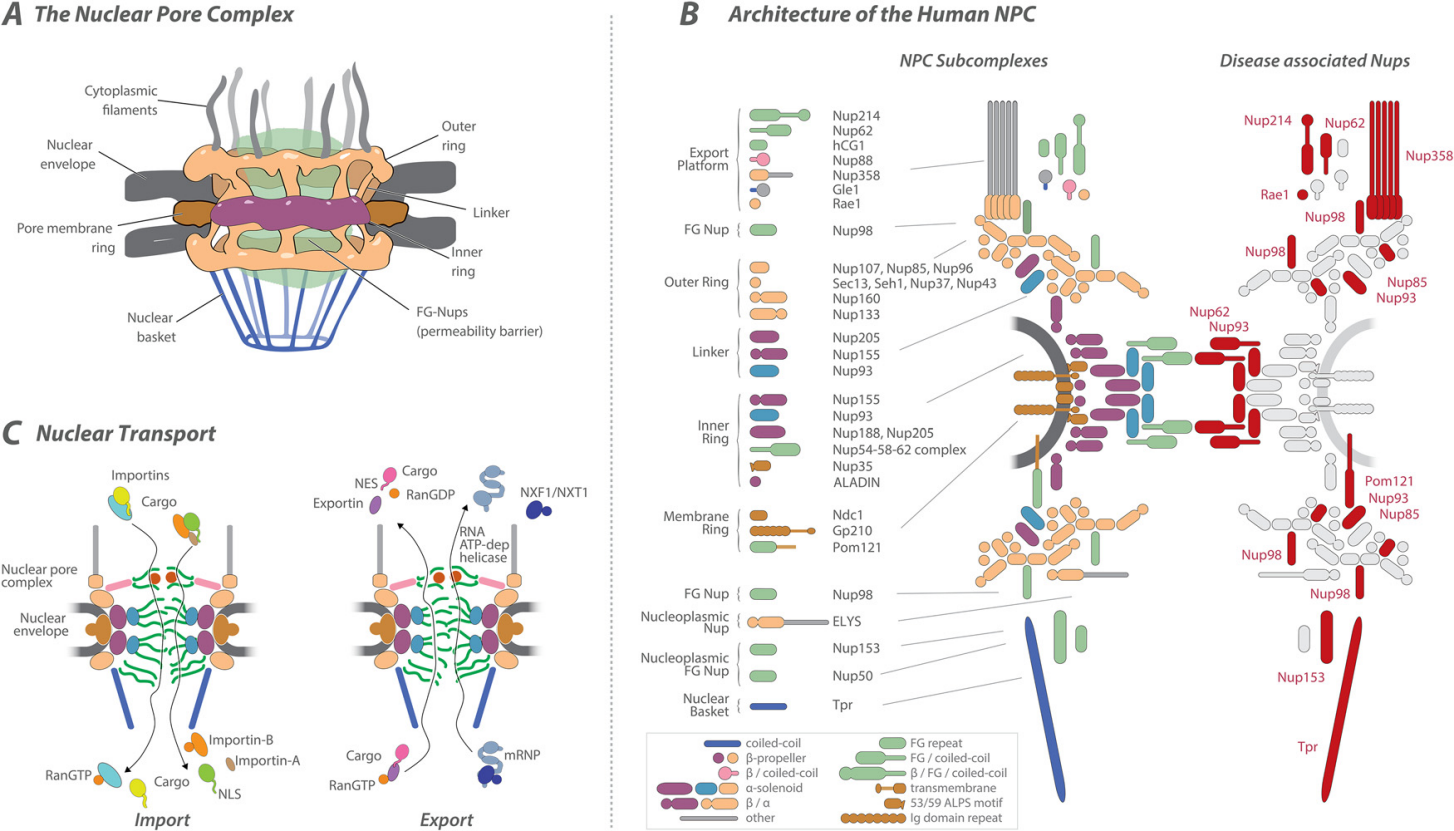
The architecture and transport function of the NPC, and virally targeted sites. (A) A cartoon of the human NPC: an 8-fold symmetric ∼110 MDa megastructure that is embedded into the nuclear envelope, with cytoplasmic filaments and a nuclear basket that mediate interactions with the cytoplasm and nucleoplasm, respectively. The cartoon is an adaptation of the illustration from NIAID NIH BioArt Source (bioart.niaid.nih.gov/bioart/000649). (B) The diagram indicates the known composition and arrangement of the different nucleoporins (Nups) in mammalian NPCs. The identity of each Nup is shown to the left. (Bottom left corner) Box depicting the protein folds and domains found in Nups; β/α indicates a β-propeller followed by an α-solenoid. Highlighted in red are the Nups targeted by viral infection. (C). Soluble factors known as karyopherins (importins and exportins) facilitate transport by binding cargoes containing nuclear export signals (NES) for export from, or NLS for import into, the nucleus. Directionality is supported by a gradient of RanGTP/GDP. Export of mRNA and associating proteins (messenger ribonucleoproteins or mRNPs) is mediated by non-karyopherin NTF2-type export factors NXF1/NXT1 that function as a heterodimer.

Though there are certainly several evolutionary pressures that shaped the emergence of the nucleus and nuclear envelope, one can speculate that viral predation was among the most powerful. By sequestering the genetic material and replication machinery behind a selective barrier, eukaryotic cells may have gained a decisive advantage over invasive genetic elements. In turn, this barrier led to viral innovation designed to regain access to host factors now confined to the nucleus. Some viruses evolved mechanisms to breach or remodel the nuclear envelope and nuclear pore complex (NPC), while others remained cytoplasmic but hijacked nucleocytoplasmic trafficking to redirect host resources toward viral replication (Figures 1 and 2).

Thus, although replication strategies differ, nearly all respiratory viruses intersect with the nucleocytoplasmic transport system, the conduit between cytoplasm and nucleus, as the nucleus is enclosed by a double-membrane nuclear envelope that is continuous with the ER, and selective exchange occurs only through NPCs. In this process, nuclear transport factors called karyopherins (importins, exportins, and transportins) and RNA transport factors bind their cargos in the cytoplasm or nucleus and ferry them across the NPC in a regulated, directionally controlled manner (Box 2). Viral strategies to breach the nucleus or hijack nucleocytoplasmic trafficking are not mutually exclusive and often disrupt both NPC structure and transport capacity. Because this system coordinates gene expression, signaling, and innate immunity, interference with it suppresses cellular defenses while reallocating resources to viral replication. Its centrality and recurrent exploitation also make it an attractive target for therapeutic modulation of nuclear transport. Cytokine and interferon (IFN)-stimulated gene networks rely on efficient nucleocytoplasmic trafficking for both signaling and effector function. Therefore, by perturbing this system without killing the host cell, viruses can selectively dampen immune defenses while redirecting transport resources to their own replication.

**BOX 2: Nucleocytoplasmic transport**

**The NPC and transport through it**

To understand exactly how these viruses control the nuclear transport machinery, we must first provide a brief refresher on the mechanism of transport and its key components. NPCs are large (∼120 MDa) macromolecular protein assemblies embedded in the NE that are the sole sites of highly selective macromolecular exchange between the nucleus and the cytoplasm (Hampoelz *et al.*, 2019; Dultz *et al.*, 2022; Stewart, 2022; Cowburn and Rout, 2023; Chen *et al.*, 2025) (Figure 2A). Each NPC contains multiple copies of ∼30 different proteins termed nucleoporins (Nups), which form an octagonal-symmetrical structure composed of eight spokes that run through the length of the NPC, totaling in humans ∼1000 proteins per NPCs (Figure 2B) (Cronshaw *et al.*, 2002; Frenkiel-Krispin *et al.*, 2010; Maimon *et al.*, 2012; Bui *et al.*, 2013; von Appen *et al.*, 2015; Hampoelz *et al.*, 2019). The NPC is anchored to the NE by a pore membrane ring, whose transmembrane domains traverse from the lumen of the NE through to the NPC and interact with a core structural scaffold (Figure 2, A and B). The core scaffold consists of two inner rings in the equatorial plane of the NPC sandwiched between outer rings that sit on the nuclear and cytoplasmic faces of the NPC. The core scaffold also anchors a class of Nups that are characterized by disordered domains that are enriched in phenylalanine glycine (FG) dipeptide repeats, termed FG-Nups and form the selective permeability barrier of the NPC by filling the central channel of the NPC (Baumann *et al.*, 2025; Chen *et al.*, 2025; Hoffmann *et al.*, 2025) (Figure 2, A and B). The barrier is entropic in nature, rather than mechanical, such that the FG repeats act as constantly moving protein strands that form a dynamic, crowded barrier—blocking random molecules while allowing transport receptors with the right cargo to pass through this milieu by virtue of rapidly interacting and handing off between successive FG repeats (Cowburn and Rout, 2023).

Although ions, metabolites, and proteins smaller than 40kDa can freely diffuse across the NPC (Feldherr and Akin, 1997; Keminer and Peters, 1999), larger protein cargos (often in complexes (Wuhr *et al.*, 2015)) require soluble transport factors collectively known as karyopherins, and also known individually as importins, exportins, and transportins (Figure 2C). Generally, karyopherins bind to structurally diverse peptide motifs termed NLSs or nuclear export sequences (NES); to mediate nucleocytoplasmic import or export through the NPC (Fornerod and Ohno, 2002; Xu *et al.*, 2012; Fung *et al.*, 2015; Wing *et al.*, 2022). In most cases, the import or export of signal-bearing cargos occurs through direct binding to their respective karyopherins (Lu *et al.*, 2021). However, the best characterized (and historically first discovered) transport pathway involves an adaptor protein forming a complex of importin α-importin β1 (Impα-Impβ) along with the cargo. In this classic pathway, Impα binds a short peptide NLS while simultaneously interacting with Impβ via its Impβ binding (IBB) domain that itself resembles an NLS (Wing *et al.*, 2022) (Figure 2C). Based on structural homology and their ability to bind to FG-Nups, most importins are classified as members of the β family of karyopherins (O'Reilly *et al.*, 2011).

The directionality of much of nucleocytoplasmic transport is governed by the small GTPase Ran, which cycles between the nucleus and the cytoplasm (Figure 2C). Ran is maintained in its GTP-bound form (RanGTP) in the nucleus by the nucleoplasmic guanine exchange factor (GEF) and in its GDP-bound form (RanGDP) in the cytoplasm by RanGAP, which stimulates GTP hydrolysis, establishing a RanGTP/RanGDP gradient across the nuclear envelope (Yang *et al.*, 2023). Importin/cargo complexes transit the NPC by transiently interacting with FG-Nups and, upon encountering Ran-GTP, on the nucleoplasmic side, are dissociated when Ran-GTP binds to Impβ. In contrast, RanGTP promotes cargo loading on exportins in the nucleus, and when, again via transient interactions with FG-Nups, this cargo-complex reaches the cytoplasmic side, RanGTP is hydrolyzed (Yang *et al.*, 2023).

Although the Ran-karyopherin transport system governs the nuclear import and export of nearly all proteins and many noncoding RNAs, including tRNA, miRNA, snRNA and rRNA, the export of mRNAs is an important exception; mRNAs, assembled into messenger ribonucleoprotein (mRNP) particles, are exported by a heterodimer composed of Nuclear Export Factor 1 (NXF1) and Nuclear Transport Factor 2 Like Export Factor 1 (NXT1) (Khan *et al.*, 2023). Directionality and energy for this process are not provided by Ran, but by the ATP-dependent helicase, DDX19, which is localized to the cytoplasmic side of the NPC and remodels mRNPs to release them from NXF1/NXT1 (Faraway *et al.*, 2025) (Figure 2C). This process is tightly coordinated with mRNP assembly, nuclear processing, and subsequent engagement with the cytoplasmic translation machinery (Fama *et al.*, 2025).

The following sections outline the major strategies respiratory viruses have evolved to subvert the nuclear transport machinery and to tilt this evolutionary arms race in their favor. These strategies, often overlapping, highlight both the nucleus's defensive origins and its continuing vulnerability to viral exploitation.

### NPC docking and nuclear entry

#### The first step—membrane penetration

During viral infections, virions enter eukaryotic cells by two major mechanisms, depending on whether they are enveloped or non-enveloped viruses (Yamauchi and Helenius, 2013). Enveloped viruses (e.g., RSV, influenza, and SARS-CoV-2) fuse directly with the cell's plasma membrane by using specific envelope proteins to engage with targeted cognate cell surface receptors (White *et al.*, 2023). Nonenveloped viruses such as adenoviruses and rhinoviruses rely on their capsid proteins to breach the plasma membrane, forming a channel that enables release of their genome directly into the cytoplasm (Pletan and Tsai, 2022). As further discussed below, typically, RNA viruses that cause respiratory disease, including the (+) strand RNA rhinoviruses, the (−) strand RNA viruses SARS-CoV-2 and RSV, replicate entirely in the cytoplasm and do not require direct access to the nucleus to replicate (Figure 1). Influenza is a notable exception. It transcribes and replicates its segmented RNA genome using its own RNA-dependent RNA polymerase within the nucleus, while hijacking host cell pre-mRNA caps as primers for viral mRNA synthesis in a process called cap snatching (Hutchinson and Fodor, 2013). On the other hand, respiratory DNA viruses, such as adenovirus, must enter the nucleus to access the host's DNA-dependent RNA polymerase for transcription of their genes, and utilize the nuclear replication machinery for their DNA synthesis (Greber and Suomalainen, 2022) (Figure 1).

#### Delivering the viral genome to the nucleus

**FIGURE 3:**
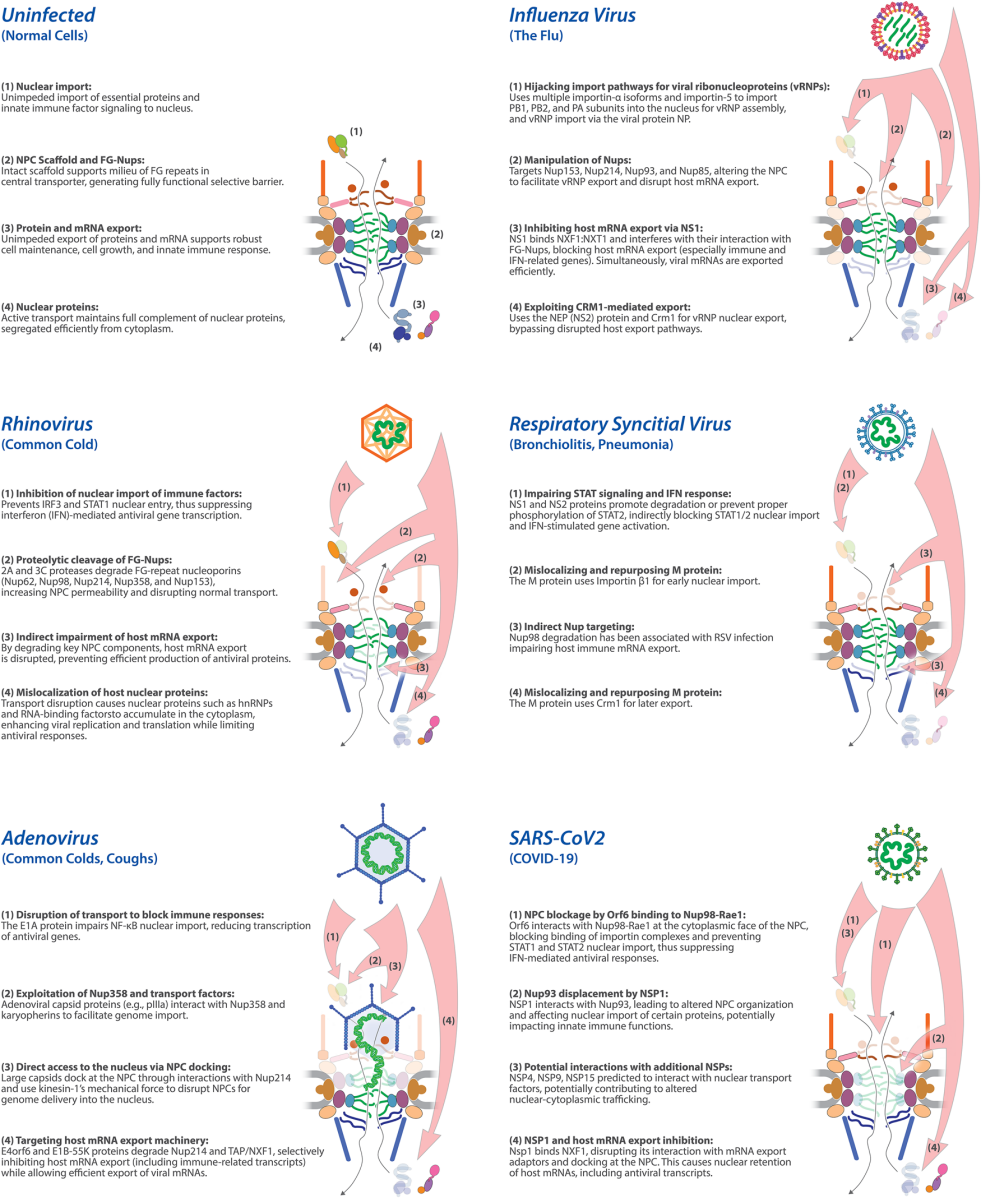
Viral strategies targeting the NPC to facilitate viral propagation. An illustration of the various strategies used by five different respiratory viruses to usurp nuclear transport. See text for details.

Depending on the virus, this involves either delivering the entire capsid or just the viral genome into the nucleus (Kobiler *et al.*, 2012). In both scenarios, interaction with the NPC is crucial for facilitating nuclear entry (Figure 1). Additionally, these viruses disrupt or co-opt the nucleocytoplasmic transport machinery to enable the trafficking of viral proteins and accessory factors between the nucleus and cytoplasm. To accomplish this, they often use a “brute force” approach—binding directly to the NPC and restructuring or dismantling it to gain nuclear access. (Figures 2 and 3).

Human adenoviruses enter eukaryotic cells via receptor-mediated endocytosis and partially disassemble and dock at the NPC through interactions of the viral hexon protein with the N-terminal domain of the host nucleoporin Nup214 to facilitate the translocation of its genome together with viral core proteins into the nucleus (Trotman *et al.*, 2001; Cassany *et al.*, 2015) (Figures 1 and 3). Once docked at the NPC, the capsid also binds to the light chains of kinesin-1, which interacts via its heavy chains with the NPC cytoplasmic filament protein Nup358 (Strunze *et al.*, 2011) (Figures 2B and 3). As a motor protein, kinesin-1 moves along the microtubules, resulting in a mechanical force both on the capsid and on the NPC, leading to capsid disassembly and NPC disruption, and increased permeability (Strunze *et al.*, 2011). The viral structural minor cement protein pIIIa directly interacts with Nup358, probably to facilitate its import into the nucleus (Ismail *et al.*, 2022) (Figures 1 and 2B). pIIIa import is critical for viral replication, capsid assembly, and DNA packaging within the nucleus (Ismail *et al.*, 2022). Although Nup358 depletion delays viral genome delivery and replication, the additional expression of karyopherin transportin-1 and, to a lesser extent, importin-9 restored the defects in genome import, indicating that nuclear transport receptors become rate-limiting under these conditions (Carlon-Andres *et al.*, 2020). It has thus been proposed that adenoviral genome delivery is promoted by an interplay between Nup358 and the transport factors, since Nup358 recruits free transport factors to the vicinity of the NPC (Carlon-Andres *et al.*, 2020) (Figure 3).

Most RNA viruses, by contrast, do not require entry into the nucleus. The notable exception is influenza. Influenza RNA is coated by nucleoprotein (NP), forming viral ribonucleoprotein complexes (vRNP) (Klumpp *et al.*, 1997) (Figure 1). NP is essential for the nuclear import of vRNP. Upon entry into cells, the influenza genome is released into the cytoplasm and imported into the nucleus by the importin Impα-Impβ heterodimer, via interaction of an NLS present on the NP (O'Neill *et al.*, 1995; Wu and Pante, 2009; Pumroy *et al.*, 2015).

### Inhibition of immune factor import

Upon viral entry, the cell's innate immune system uses a variety of defense mechanisms to combat infection. These defenses are activated upon detection of viral components and function to inhibit viral replication, initiate adaptive immune responses, and even promote the autodestruction and clearance of infected cells. Primary among these responses is the production of interferons (IFNs) (de Veer *et al.*, 2001). IFNs are secreted cytokines that trigger the rapid transcription of hundreds of IFN-stimulated genes (ISGs), which collectively inhibit viral replication at multiple stages, regulate inflammation and cellular stress, and render the cell inhospitable to viruses (de Veer *et al.*, 2001). Upon recognizing characteristic pathogen-associated molecular patterns (PAMPs) in viral associated molecules—for example, nucleic acids such as 5′-phosphorylated RNA and dsRNAs—various receptors, including Toll-like receptors (TLRs), RIG-I–like receptors (RIG-I), and cyclic GMP–AMP synthase (cGAS) (Zevini *et al.*, 2017; Kawai *et al.*, 2024) activate the Janus kinase/signal transducer and activator of transcription (JAK/STAT) signaling cascade, leading to the phosphorylation and activation of STAT1 and STAT2 (Au-Yeung *et al.*, 2013; Steen and Gamero, 2013; Rincon-Arevalo *et al.*, 2022). The STAT1-STAT2 heterodimer associates with IRF9, and this complex is imported into the nucleus by Impα-Impβ and binds to certain IFN-stimulated response elements to activate the transcription of ISGs. As a central antiviral hub, this pathway is therefore a prime target for viral countermeasures, which frequently block or misroute nuclear import to suppress ISG induction.

#### SARS-CoV-2 Orf6 at the NPC blocks STAT import

SARS-CoV-2 Orf6 localizes to the cytoplasmic face of the NPC by interacting with the NPC (Makio *et al.*, 2024) (Figure 3). Its C-terminal domain interacts with Nup98-Rae1 (Gordon *et al*., 2020) and impairs ISG induction by blocking STAT1 and STAT2 nuclear translocation, likely by disrupting Nup98 interactions with Impα-Impβ (Miorin *et al.*, 2020). Orf6 positioning within the NPC may also disrupt FG-Nup interactions with nuclear transport factors and thus their ability to support nuclear transport, including that of STAT1 and STAT2 (Miorin *et al.*, 2020; Makio *et al.*, 2024). Interestingly, Orf6 can also bind STAT1 directly and interact with importin α subtypes, with a particularly high affinity for importin α1, suggesting that there is greater complexity in Orf6 interactions than is currently understood (Miyamoto *et al.*, 2022). Because Rae1 depletion suppresses Orf6-mediated inhibition of p-STAT1 import, it appears to play a dominant role in disrupting transport at the NPC (Addetia *et al*., 2021). The related SARS-CoV Orf6 binds Nup98–Rae1 with weaker phenotypes (Addetia *et al*., 2021; Gao *et al*., 2022).

#### Further disruption of nuclear transport by SARS-CoV-2

Protein-protein interaction mapping of SARS-CoV-2 predicted that additional SARS-CoV-2–encoded proteins, Nsp4, Nsp9, and Nsp15, also associate with the host nuclear transport machinery; however, these interactions and their effect have not yet been validated (Gordon *et al.*, 2020). Nonetheless, SARS-CoV-2 protein Nsp1 has been shown to interact with Nup93 (Gomez *et al.*, 2019; Yuan *et al.*, 2021). Nup93 is located in the NPC's inner ring and serves as a linker between three FG-Nups (Nup54, Nup58, and Nup62) and the core scaffold (Figure 2B). Nup93-Nsp1 interaction leads to disrupted localization of Nup93 that, in turn almost certainly leads to altered nucleocytoplasmic transport and altered permeability, given that this protein acts as a keystone in the NPC's scaffold (Kim *et al.*, 2018). Specifically, nucleolin, a multifunctional nucleolar protein, accumulates in the cytoplasm in the presence of Nsp1, suggesting its nuclear import is disrupted (Gomez *et al.*, 2019). Interestingly, although nucleolin import is disrupted, there is no global effect on nuclear import, suggesting that the NPC permeability barrier is maintained, while only a specific set of import cargo is affected. Because Nup93 plays a role in mediating antiviral innate immune response (Monwan *et al.*, 2020), its displacement by Nsp1 likely alters both the nucleocytoplasmic transport and perturbs the innate immune response (Figure 3).

#### RSV blocks STAT import and IRF3 activation

The RSV-encoded proteins NS1 and NS2 promote proteasomal degradation of STAT2, thereby blocking its nuclear import and dampening IFNα/β signaling (Lo *et al.*, 2005; Elliott *et al.*, 2007). Silencing NS1/NS2 accordingly elevates IFN-responsive transcripts, including IFN-α, IFN-β, and IFN-λ2/3 (Spann *et al.*, 2004). NS2 is a multifunctional IFN antagonist that targets both induction and signaling pathways (Ling *et al.*, 2009) (Figure 3). RSV also targets the RIG-I/MDA5–MAVS axis to impair IRF3 activation and nuclear translocation, key elements of the host's antiviral defenses (reviewed in Sedeyn *et al.* (2019). Specifically, NS1 blocks the interaction between RIG-I and MAVS and IRF3 phosphorylation (Ban *et al.*, 2018), and NS2 binds directly to RIG-I, disrupting the formation of the IRF3/CREB-binding protein complex, further impairing IRF3’s activity (Spann *et al.*, 2004; Spann *et al.*, 2005). Similarly, Influenza A virus NS1 inhibits IRF3 activation (Talon *et al.*, 2000) (Figure 3).

### Hijacking nuclear protein transport and karyopherins

Exploiting or repurposing specific karyopherins is a common strategy by which viruses manipulate host nuclear transport. Some viruses actively hijack transport receptors, binding them directly or mimicking host cargo, while others passively depend on normal receptor function to shuttle viral components or deplete transport capacity for host factors. Both are ways to circumvent the selective barrier imposed by the nuclear envelope and NPC.

#### RSV M protein

The RSV M protein simply jumps aboard the transport system, using Importin β1 for nuclear import early in infection, where it is thought to regulate host transcription, and later relying on Crm1 for export to the cytoplasm, a step essential for virus assembly (Ghildyal *et al.*, 2005; Ghildyal *et al.*, 2009) (Figure 3). Pharmacological inhibition of Crm1 traps M in the nucleus, impairing viral replication and highlighting the virus's reliance on this host export pathway (Ghildyal *et al.*, 2009; Jorquera *et al.*, 2019; Mathew *et al.*, 2021).

#### Influenza virus polymerase and NP transport

Influenza virus NPs and polymerase complexes (PB1, PB2, and PA) must enter the nucleus for viral RNA synthesis (Ling *et al.*, 2022). To accomplish this, they have evolved nuclear localization signals (NLS) that allow access to the karyopherin family for nuclear import (Ozawa *et al.*, 2007; Nguyen *et al.*, 2023). Newly produced polymerase subunits rely on importin 5 (for PA and PB1) and multiple Impα isoforms (Impα1, Impα3, Impα5, and Impα7) to import PB1 (Huet *et al.*, 2010; Li *et al.*, 2015). PB1 and PB2 interact with Nup85 that facilitates their binding to importin-5, importin α1, and importin α7 (Ling *et al.*, 2022) (Figures 2C and 3). This prioritizes the nuclear import of viral ribonucleoproteins, overwhelming the nuclear import machinery and reducing the efficiency of nuclear import for immune signaling factors such as STATs and IRFs (Mukaigawa and Nayak, 1991). The NPC nuclear basket-associated protein Nup50 may further stabilize PB2 in the nucleus by preventing its rebinding to Importin α5 (Pumroy *et al.*, 2012).

To exit the nucleus, influenza encodes a nuclear export protein (NEP, formerly termed NS2) that binds viral ribonucleoproteins and carries a nuclear export sequence that binds to the nuclear export factor, Crm1 (Paterson and Fodor, 2012; Huang *et al.*, 2013). This ensures efficient Crm1-dependent export of viral genomes to the cytoplasm for packaging into progeny virions.

### Sabotage of host mRNA export

Viral control of mRNA export provides a complementary tactic to establish a coordinated strike on nuclear transport, again controlling resources and the cell's defenses. This control usually has two objectives, namely, to simultaneously suppress host mRNA export, favoring viral mRNA export and protein production, while minimizing the export of mRNAs involved in innate immune responses. Given the potential effectiveness of this strategy, it is often one used to a major extent by many viruses.

#### SARS-CoV-2: Nsp1 and Orf6 rewire export

SARS-CoV-2 Nsp1 disrupts the host mRNA export machinery by binding the NXF1:NXT1 nuclear export receptor complex, interfering with its association with the mRNA export adaptors Aly/REF and the ATP-dependent helicase UAP56 and its docking at the NPC (Zhang *et al.*, 2019; Vasudevan and Baraniuk, 2021; Mei *et al.*, 2024) (Figure 3). Accordingly, Nsp1 expression in cells decreases host mRNA levels and disrupts overall mRNA export, leading to the accumulation of mRNA in the nucleus (Guillen and Glaunsinger, 2024).

SARS-CoV-2 Orf6 acts through a complementary mechanism, as discussed above; it engages the Nup98:Rae1 complex, disrupting host nuclear transport and impairing NPC function, which induces the nuclear accumulation of host mRNAs and RNA-binding proteins such as hnRNPA1 (Addetia *et al.*, 2021; Kato *et al.*, 2021; Hall *et al.*, 2022). Although Rae1 has been reported to bind mRNA and the mRNA export factor TAP/NXF1 (Bachi *et al.*, 2000; Blevins *et al.*, 2003), Rae1 depletion does not impair bulk poly(A)+ RNA export, suggesting that Orf6 expression imposes selective export blockades rather than a general RNA export disruption (Makio *et al.*, 2024). Notably, Rae1 is nevertheless essential for SARS-CoV-2 protein production during infection, suggesting that the virus repurposes this complex for its own gene expression while restricting host export (Makio *et al.*, 2024).

#### Influenza NS1 coordinates viral mRNA export while blocking host export

Influenza uniquely replicates its RNA genome in the nucleus, coupling viral mRNA production to nuclear export. The viral Non-structural protein NS1 acts as a multifunctional regulator of host RNA metabolism. NS1 binds the cellular mRNA export receptor heterodimer NXF1:NXT1, promoting export of viral transcripts such as the unspliced M1 mRNA (encoding the most abundant viral protein) and its spliced variant M2, which encodes an ion channel (Pereira *et al.*, 2017). Simultaneously, this hijacking of the export complex inhibits host mRNA export.

Mechanistically, NS1 interferes with NXF1:NXT1 interactions with FG-Nups, particularly those Nups forming the cytoplasmic export platform (Zhang *et al.*, 2019) (Figure 2). Immune-related transcripts, including ISGs, are among the most retained in the nucleus following infection (Zhang *et al.*, 2019), underscoring NS1’s dual function in diverting export resources and disrupting defenses.

NS1 also forms a complex with NXT1, Rae1, E1B-AP5, and Nup98 (Satterly *et al.*, 2007), thereby further inhibiting the canonical mRNA export route (Figure 3). In addition to Nup98, influenza proteins interact with several nucleoporins involved in mRNA export, including Nup214 (Senbas Akyazi *et al.*, 2020), Nup153 (Muhlbauer *et al.*, 2015), Nup93 (Furusawa *et al.*, 2018), and Nup85 (Ling *et al.*, 2022), a core scaffold nucleoporin that anchors the cytoplasmic mRNA export platform (Nup88 complex) (Figure 2B). Although Nup214, Nup153, and Nup98 are directly involved in mRNA trafficking, disruption of structural components like Nup85 and Nup93 also perturbs NPC integrity and transport function. Likewise, perturbing Nup93, which links FG-Nups (Nup54 and Nup62) to the core scaffold, is likely to further broaden transport defects (Figure 3).

Despite this host blockade, as mentioned above, influenza ensures the efficient export of its own viral ribonucleoproteins (vRNPs) through the viral nuclear export protein NEP, which binds viral RNA and carries a Crm1-recognized nuclear export sequence (Neumann *et al.*, 2000; Iwatsuki-Horimoto *et al.*, 2004; Pereira *et al.*, 2017). This Crm1-dependent route bypasses host inhibition, allowing coordinated release of viral genomes for packaging and budding.

#### Adenovirus: selective host shutoff with viral mRNA export

In the late phase of adenovirus infection, viral mRNAs are efficiently exported from the nucleus to the cytoplasm, whereas most host cellular mRNAs remain trapped in the nucleus (Weigel and Dobbelstein, 2000). This selectivity is accomplished by the action of two viral proteins forming the E1B-55K/E4orf6 complex, which acts as an E3 ubiquitin ligase targeting host export factors (Bridge and Ketner, 1989; Blanchette *et al.*, 2008). E4orf6 recruits and relocalizes E1B-55K from the cytoplasm to the nucleus (Goodrum *et al.*, 1996), and the complex shuttles continuously between the nucleus and cytoplasm, likely through an NES within E4orf6 (Weigel and Dobbelstein, 2000). The result is a reengineered export environment that suppresses host mRNA export, including transcripts that encode cytokines and IFNs, while promoting the nuclear export of viral late mRNAs (Figure 3).

### Degradation of FG-Nups

In addition to directly binding to NPC components, some viruses strategically take out key Nups positioned in the NPC to disrupt nucleocytoplasmic transport and to diminish or alter the permeability barrier of the NPC. Typically, this is mediated by either viral proteases or by recruitment of host factors such as caspases (Ferrando-May *et al.*, 2001; Muhlbauer *et al.*, 2015); all of which is again to provide the virus greater access to the replicative resources, while impairing the cell's ability to mount an effective response to infection.

#### FG-Nup degradation by picornaviruses/rhinoviruses

Picornaviruses, of which the respiratory disease rhinoviruses are a member, degrade key FG-Nups (Nup62, Nup98, Nup214, Nup358, and Nup153) via viral proteases 2Apro and 3Cpro (Gustin and Sarnow, 2002; Park *et al.*, 2008; Walker *et al.*, 2013; Serganov *et al.*, 2022) (Figures 2B and 3). None of the structural Nups are degraded by these proteases, and so the overall structural integrity of the NPC is maintained while its selective transport ability is badly damaged. This FG Nup degradation leads to increased permeability of the NPC (Park *et al.*, 2015), resulting in the diffusion into the nucleus of large normally cytoplasmic molecules that lack nuclear targeting sequences (Jensen *et al.*, 2015). Early in infection, 2Apro primarily targets Nup98, with only a marginal effect on nuclear import, while nuclear export of proteins and mRNAs is more strongly affected, likely to impede innate signaling (Serganov *et al*., 2022). As infection progresses, and more FG-Nups are lost, import also fails as receptor-cargo complexes fail to dock at the NPC (Gustin and Sarnow, 2002) and nuclear proteins, including proviral factors, are relocalized to the cytoplasm (Gustin and Sarnow, 2002; Walker *et al*., 2013; Flather *et al*., 2018). Influenza likewise induces Nup98 degradation to suppress transport, most obviously at the level of mRNA export (Satterly *et al.*, 2007).

#### Caspase-driven Nup cleavage

Apoptosis, the programmed cell death pathway, is a frequent consequence of viral infection (Verburg *et al.*, 2022). Often, viruses attempt to modulate apoptosis sufficiently to ensure their replication home remains mostly intact, but the targeting of Nups in this process can also be exploited to favor their replication (Herold *et al.*, 2012). A hallmark of apoptosis is the activation of caspases, a family of cysteine proteases that orchestrate the dismantling of cellular components (Kopeina *et al.*, 2018). Among their targets are several nucleoporins, including Nup153, Nup358, Nup214, TPR, and POM121, contributing to the breakdown of nuclear transport (Ferrando-May *et al.*, 2001; Kopeina *et al.*, 2018) (Figure 2B). Influenza infection, for example, induces caspase-mediated degradation of Nup153 (Muhlbauer *et al.*, 2015), resulting in an enlargement of the NPC and weakening its permeability barrier. Consequently, passive export of viral RNPs increases, reducing dependency on host transport factors (Muhlbauer *et al.*, 2015).

### Exploiting mitotic nuclear envelope breakdown

Some viruses exploit the transient loss of nuclear compartmentalization that occurs during mitosis (Hetzer *et al.*, 2005). During the prophase and prometaphase, phosphorylation of nuclear lamins and nuclear pore complex (NPC) components dismantles the NPC and nuclear envelope, allowing mitotic spindle microtubules to access chromosomes and drive cell division (Heald and McKeon, 1990; Burke and Ellenberg, 2002; Glavy *et al.*, 2007; Laurell *et al.*, 2011). Human adenovirus 5 (HAd5) exploits this opportunity to inhibit STING activity through its viral oncoprotein E1A (Lau *et al.*, 2015), illustrating how viral timing intersects with mitotic remodeling to outmaneuver host defense systems.

#### Perspectives

We propose that the nucleus evolved, at least in part, to shield the cell's DNA replication machinery from viral attack. Yet evolution is an arms race: although many viruses adapted to replicate entirely in the cytoplasm, others evolved arcane and ingenious mechanisms to reach or exploit the nucleus and target the nuclear transport machinery with striking precision.

Respiratory viruses such as adenoviruses, rhinoviruses, influenza, and SARS-CoV-2 exemplify this convergence at the transport machinery, all targeting nuclear transport to gain control. They hijack nuclear transport factors, dismantle FG-Nups, block immune signaling, and reroute mRNA export. Some physically breach the NPC to deliver their genomes, others remain cytoplasmic yet selectively silence specific aspects of the host's communication between the nucleus and cytoplasm.

These strategies reveal both the adaptability of viruses and the central role of nuclear transport in host cell defense and maintenance. Recognizing and understanding this complex interplay illuminates new therapeutic opportunities. Many human viruses still lack effective treatments, and new viral threats continue to emerge (Howard and Fletcher, 2012; Wang *et al.*, 2021). Traditional antivirals often target viral proteins prone to rapid mutation and resistance. On the other hand, host-directed therapy can exploit the stable dependencies viruses create, and the nuclear transport machinery appears to provide ample targets. But nuclear transport is also a daunting target, because it is essential to all cells. As outlined here, the essential nuclear transport system becomes rewired and stressed upon viral infection, operating at a subcritical level, ensuring the host cell can survive long enough to support virus production. But what if, based on the principle of synthetic lethality, we could tip the system over the edge? Synthetic lethality exploits the fact that when viruses rewire host networks to favor their replication, they create dependencies that do not exist in uninfected cells (Mast *et al.*, 2020; Navare *et al.*, 2022; Pal *et al.*, 2022). By disrupting host genes in ways that are nonessential in healthy cells but become essential in infected ones, therapies can selectively kill or disable infected cells. This shifts the drug target from the genetically volatile virus to much more stable host factors, lowering the risk of resistance and opening the door to for pan-viral strategies, since diverse viruses converge on a very similar way to subvert nuclear transport. CRISPR-based screens have already begun to uncover such infection-specific vulnerabilities, many of which are synthetically lethal with nuclear transport (Staheli *et al.*, 2024). Combining judicious inhibition of hijacked nuclear transport with synthetic lethal targeting of virus-induced host dependencies creates a two-pronged strategy: inhibiting the routes viruses use and exploiting the weaknesses they expose. What began as a defensive cellular innovation over two billion years ago may yet be exploited to provide a new kind of immunity—engineered this time, not by evolution, but by human design.

## Abbreviations used:

2A/3Cpro: 2A/3C protease
5′-UTR - 5′: Untranslated Region
Aly/REF: Aly/REF export factor
cGAS: Cyclic GMP-AMP synthase (guanosine monophosphate–adenosine monophosphate)
CREB: cAMP-responsive element-binding protein
CRISPR: Clustered Regularly Interspaced Short Palindromic Repeats
Crm1: Chromosome region maintenance 1 protein (Exportin 1)
DDX19: DEAD-Box Helicase 19
DNA: Deoxyribonucleic Acid
dsRNA: double stranded Ribonucleic acid
E1A: Adenovirus Early Region 1A
E1B-AP5: Adenovirus Early Region 1B-associated protein 5
E1B-55K: Adenovirus Early Region 1B 55-kilodalton protein
E4orf6: Early Region 4 Open Reading Frame 6
ER: endoplasmic reticulum
FG-Nup: Phenylalanine-Glycine Nucleoporins
GDP/GTP: Guanosine Diphosphate/Guanosine Triphosphate)
GEF: guanine exchange factor
hAdV: human adenovirus
IBB: Impβ binding domain
IFN: interferon
Imp α/β: importin α/β
IRF3/IRF9: Interferon Regulatory Factor 3/9
ISG: interferon stimulated gene
JAK/STAT: Janus kinase/signal transducer and activator of transcription
mRNPs: messenger ribonucleoproteins
M protein: matrix protein
MAVS: Mitochondrial AntiViral-Signaling protein
MDA5: Melanoma Differentiation-Associated protein 5
mRNA, tRNA, miRNA, snRNA and rRNA: messenger, transfer, micro, small nuclear, ribosomal Ribonucleic acid
NEP: nuclear export protein
NES: nuclear export signal
NIAID: National Institute of Allergy and Infectious Diseases
NIH: National Institutes of Health
NLS: nuclear localization signal
NP: nucleoprotein
NPC: Nuclear pore complex
NS: nonstructural
Nsp: Nonstructural protein
NTF2: Nuclear transport factor 2
Nup: Nucleoporin
NXF1: Nuclear RNA export factor 1
NXT1: NTF2-related export protein 1
PA: Polymerase Acidic
PAMPs: pathogen associated molecular patterns
PB1/PB2: Polymerase Basic 1/2
POM121: Pore Membrane protein of 121 kDa (NPC component)
Rae1: Ribonucleic acid export 1)
RIG-I: Retic acid-Inducible Gene-I
RLRs: RIG-I-like receptors
RNA: Ribonucleic acid
RNP: Ribonucleoprotein
RSV: Respiratory syncytial virus
SARS-CoV-2: Severe Acute Respiratory Syndrome Coronavirus 2
STAT: Signal transducer and activator of transcription
STING: Stimulator of Interferon Genes
TAP: Transcriptional Activating Protein
TLRs: Toll-like receptors
TPR: Translocated Promoter Region (NPC basket protein)
UAP56: U2AF65-associated protein, 56 kDa (RNA helicase DDX39B)
VP: viral coat protein
vRNP: viral Ribonucleoprotein.
